# The Molecular Neuroimaging of Tremor

**DOI:** 10.1007/s11910-021-01157-4

**Published:** 2021-11-24

**Authors:** Jacopo Pasquini, Roberto Ceravolo

**Affiliations:** 1 Department of Pathophysiology and Transplantation, University of Milan, Milan, Italy; 2 Clinical Ageing Research Unit, Newcastle University, Campus for Ageing & Vitality, Westgate Road, Newcastle upon Tyne, NE4 5PL UK; 3 Department of Clinical and Experimental Medicine, University of Pisa, Pisa, Italy; 4 Neurodegenerative Diseases Center, Azienda Ospedaliero Universitaria Pisana, Pisa, Italy

**Keywords:** Tremor, Molecular neuroimaging, Parkinson’s disease, Essential tremor

## Abstract

**Purpose of Review:**

Tremor is a hyperkinetic movement disorder most commonly encountered in essential tremor (ET) and Parkinson’s disease (PD). The purpose of this review is to summarize molecular neuroimaging studies with major implications on pathophysiological and clinical features of tremor.

**Recent Findings:**

Oscillatory brain activity responsible for tremor manifestation is thought to originate in a cerebello-thalamo-cortical network. Molecular neuroimaging has helped clarify metabolic aspects and neurotransmitter influences on the main tremor network. In ET, recent positron emission tomography (PET) studies are built on previous knowledge and highlighted the possibility of investigating metabolic brain changes after treatments, in the attempt to establish therapeutic biomarkers. In PD, molecular neuroimaging has advanced the knowledge of non-dopaminergic determinants of tremor, providing insights into serotonergic and noradrenergic contributions.

**Summary:**

Recent advances have greatly extended the knowledge of tremor pathophysiology and it is now necessary to translate such knowledge in more efficacious treatments for this symptom.

## Introduction

Tremor is an involuntary, rhythmic, oscillatory movement of a body part. According to the latest consensus statement on the classification of tremor of the International Parkinson and Movement Disorder Society, tremor may be classified along two axes, i.e. clinical features (axis 1) and etiology (axis 2) [1]. The most common tremor etiologies are essential tremor (ET) and parkinsonian tremor. While ET is classically characterized by bilateral upper limb postural and kinetic (i.e. action) tremor without other neurological signs, parkinsonian tremor is classically at rest and accompanied by bradykinesia. Of note, segregation between essential tremor and parkinsonian tremor is not always clinically obvious, as essential tremor patients may present additional mild neurological signs, such as a rest tremor component, dystonic posturing, difficult tandem walking etc., a phenotype named ET plus. Furthermore, ET patients may also have an increased risk of developing Parkinson’s disease (PD); this association has been thoroughly reviewed in [2]. From a neurophysiological point of view, parkinsonian rest tremor is typically a 4–6-Hz tremor, while action tremor of ET is a 5–8-Hz tremor [3]. Other, less frequent forms of tremor include dystonic tremors, characterized by the association with dystonia, and Holmes tremor, usually associated with other brainstem or thalamic localizing signs.

In this review, we will highlight the pathophysiological and clinical aspects of tremor as investigated through molecular neuroimaging, such as positron emission tomography (PET) and single-photon emission tomography (SPECT). We will also refer non-systematically to other techniques, such as magnetic resonance imaging (MRI) and neurophysiological studies of tremor. The major focus of this review will be on ET and parkinsonian tremor. Brief paragraphs will be dedicated to dystonic tremor and Holmes tremor.

## Search Strategy

Pubmed/MEDLINE database was interrogated. Search words were “tremor” and: “molecular neuroimaging”, “single photon emission tomography”, “positron emission tomography”. Articles written in English investigating tremor in humans through molecular neuroimaging were selected; additional articles with these characteristics were extracted from the references.

## Molecular Neuroimaging Correlates of Essential Tremor

The mechanism of tremor generation in ET is only partially understood and thought to be due to a network dysfunction involving the cerebellum, thalamus, motor cortex and perhaps the inferior olivary complex and the red nucleus [4]. Furthermore, ET itself is a dynamic disease and it is currently debated whether it is only characterized by an abnormal activity in the tremor network or it is part of a broader neurodegenerative spectrum [5, 6]. The current main medication available for ET are beta-blockers, such as propranolol, and GABA-enhancing drugs such as primidone, benzodiazepines and gabapentin [7]. Different mechanisms and neurotransmitter systems influence the main tremor network and molecular imaging approaches have been used to investigate pathophysiological aspects of ET. Molecular neuroimaging approaches in essential tremor have explored cerebral blood flow, metabolism and neurotransmitter binding sites.

### Cerebral Blood Flow and Metabolism in Essential Tremor

Early studies mostly focused on investigating alterations of cerebral blood flow in various brain regions included in the tremor network. To investigate regional blood flow, PET studies used radiolabelled water (e.g. H_2_^15^O) or carbon dioxide (C^15^O_2_) and SPECT studies used ^99m^Tc-HMPAO or ^99m^Tc-ethyl-cysteinate dimer (^99m^Tc-ECD). Initially, the rationale of studying the tremor network through regional cerebral blood flow was to establish the central origin of essential tremor and to study its cerebral correlates. Of note, these studies included no more than 11 patients and 10 controls each. A common finding in ET was a bilateral increase in cerebellar regional blood flow, indicating increased cerebellar activity, at rest and when posturing compared to controls [8–12]. Notably, it was also shown that bilateral increase of cerebellar blood flow was observed only in ET while posturing (“tremor task”) but not with passive flexion–extension of the wrist at tremor frequency (“proprioceptive task”), indicating that cerebellar activation is related to tremulous activity and not to a proprioceptive feedback. One study that employed a wider field of view also found significant increases in activity of the red nuclei and thalami during tremulous activity in patients with ET [10].

Glucose metabolism has also been explored, although to a lesser extent. One of the earliest ^18^F-fluorodeoxyglucose (FDG) PET studies reported an increase in medullary and thalamic, but not cerebellar, glucose metabolism in patients with ET compared to controls, suggesting an important role of the inferior olivary nucleus in generating tremor in ET [13]. More recent studies highlighted reductions in cortical frontal, temporal, precuneus and occipital metabolism in ET patients compared to controls, while they showed heterogeneous results regarding cerebellum glucose metabolism [14, 15]. Recently, two studies have employed FDG-PET to monitor regional glucose metabolism changes after interventions for ET. In one study, FDG-PET was conducted before and after gamma knife surgery of the ventral intermediate nucleus (Vim) of the thalamus in 42 ET patients. More than 1 year after surgery, a significant decrease in glucose metabolism was found within the left thalamus, the right cerebellum and frontal and temporal areas that were identified as hypermetabolic compared to controls before treatment [16•]. Furthermore, decreased temporo-occipital metabolism at baseline was predictive of a lack of tremor response after surgery. This study highlighted the remote effect of a lesion inside the tremor network and the possibility of investigating metabolic biomarkers that may help predict the response to treatments. Another small pilot trial measured brain metabolic activity with FDG-PET in 5 ET patients enrolled in an open-label trial with transcutaneous afferent patterned stimulation (TAPS) [17•], a non-invasive electrical stimulation of peripheral nerves at the wrist known to reduce hand tremor in ET [18]. Reduction in tremor amplitude was on average 73% after treatment. After 90 days of treatment, compared to baseline, FDG-PET revealed increased metabolism in the cerebellar hemisphere ipsilateral to the stimulation and hypometabolism contralaterally, and a moderate-strong correlation was found between the change in tremor severity and the change in FDG uptake in the hypermetabolic hemicerebellum. Some cortical areas, including frontal, occipital and parietal showed decreased metabolism after treatment. The reduction of metabolism in some cortical areas and in one cerebellar hemisphere seems consistent between these two interventional studies [16•, 17•]. Therefore, FDG-PET appears an appropriate technique to investigate cerebral metabolic changes after interventions for ET and future studies are needed to investigate metabolic correlates of ET treatments that could yield potential disease and therapeutic biomarkers.

### Receptor and Neurotransmitter Imaging in Essential Tremor

Receptor imaging in ET has mostly been focused on dopaminergic pathways. Studies were initially conducted to explore the relationship between ET and PD and have provided a great wealth of data about the pathophysiology of tremor in these conditions. The majority of these studies did not find significant reductions in striatal dopaminergic markers in patients with a classic essential tremor presentation compared to controls (Table 1) [19–27]. Conversely, dopamine transporter imaging in patients with atypical tremor (e.g. unilateral presentation or presence of a rest component) and/or additional “soft” parkinsonian signs (e.g. mild rigidity or bradykinesia) highlighted a blurred territory between ET and PD. Indeed, some patients with atypical features and additional parkinsonian signs may demonstrate striatal DaT reductions comparable to those with PD and usually convert to overt PD after a few months or years, while patients with normal striatal DaT will retain their tremulous phenotype over the same period of time (Table 2) [19, 28–33]. These studies highlight the heterogenous nature of the underlying pathophysiological process in patients presenting with mixed and atypical tremor presentations. However, while reduced striatal DaT represents a biomarker of PD in these patients, it is currently unknown whether patients with atypical tremors and normal striatal DaT have an increased risk of developing PD or other neurodegenerative diseases in the longer term. In the future, large longitudinal studies with a detailed phenotyping are needed to clarify whether patients with ET have an increased risk of developing PD and whether any possible associated risk factors and biomarkers can be identified.

**Table 1:** Summary of selected studies investigating the state of the nigro-striatal dopaminergic markers in essential tremor and Parkinson’s disease

Author, year	Technique	Cohort	Findings
Brooks et al. (1992) [19]	^18^F-DOPA PET	20 isolated PT 11 isolated RT 16 PD 20 HCs	2 of 20 isolated PT had subnormal putamen ^18^F-DOPA activity, one of whom in the PD range and later became akinetic. 18 of 20 had normal tracer activity All isolated RT patients had putamen ^18^F-DOPA activity in the PD range
Asenbaum et al. (1998) [26]	^123^I-β-CIT SPECT	32 ET (without soft signs) 29 PD (HY stage 1) 30 HCs	ET: normal striatal uptake compared to controls PD: reduced striatal uptake compared to controls
Benamer et al. (2000) [21]	^123^I-FP-CIT SPECT	27 ET 158 parkinsonism 35 HCs	ET: 25 of 27 scans visually graded as normal Parkinsonism: 150 of 158 visually graded as abnormal HC: 33 of 35 visually graded as normal
Parkinson Study Group (2000) [20]	^123^I-β-CIT SPECT	14 ET 43 PD 17 PSP 22 HCs	Visual inspection agreed with clinical diagnosis in: 98% of parkinsonism, 83% of ET/HCs Quantitative analysis did not find differences between ET and HCs (but found lower uptake in ET patients visually diagnosed as parkinsonism)
Isaias et al. (2010) [24]	^123^I-FP-CIT SPECT ^99^Tc-ECD SPECT (prefusion imaging)	20 ET 13 PD 23 HCs	ET: normal striatal FP-CIT uptake at baseline and after 3 years. Some ET patients have caudate uptake lower than controls Tremor network analysis with perfusion imaging revealed significant and discriminant differences between ET and PD
Roselli et al. (2010) [25]	^123^I-FP-CIT SPECT (dopamine and serotonin transporter imaging)	11 ET 15 PD, 16 LBD, 10 PSP 9 HC	ET: normal striatal and midbrain raphe FP-CIT uptake PD: reduced striatal and normal midbrain raphe FP-CIT uptake
Di Giuda et al. (2012) [23]	^123^I-FP-CIT SPECT	15 ET 21 PD 17 HCs	ET: normal striatal uptake. Severity of anxiety symptoms inversely correlated with left caudate DAT PD: caudate DAT inversely correlated with depressive symptoms

Abbreviations:* ET*, essential tremor; *HC*, healthy controls; *HY*, Hoehn and Yahr scale: *PET*, positron emission tomography; *PD*, Parkinson’s disease; *PT*, postural tremor; *RT*, rest tremor; *SPECT*, single-photon emission computed tomography

Radioligands: ^18^F-DOPA: ^18^F-fluoro-L-dopa; ^123^I-beta-CIT SPECT: ^123^I-2β-carbomethoxy-3β-(4-iodophenyl)tropane; ^123^I-FP-CIT SPECT: ^123^I-N-3-fluoropropyl-2β-carboxymethoxy-3β-(4-iodophenyl) nortropane

**Table 2:** Summary of selected studies investigating the state of the nigro-striatal dopaminergic markers in patients with a tremor presentation and atypical features (e.g. unilateral or markedly asymmetrical presentation) and/or additional “soft” parkinsonian signs (e.g. rest tremor, mild rigidity or bradykinesia)

Author, year	Technique	Cohort	Findings
Lee et al. (1999) [27]	^123^I-IPT SPECT	9 isolated PT 6 PT and RT 11 PD 21 HCs	Isolated PT: normal striatal uptake PT and RT: uptake significantly lower than HCs and similar to PD PD: reduced striatal uptake
Schwartz et al. (2004) [28]	^123^I-FP-CIT SPECT	10 ET with impairment at visuo-motor testing	Decreased striatal uptake in 8 of 10 patients. Striatal uptake correlated with visuo-motor testing score
Ceravolo et al. (2008) [29]	^123^I-FP-CIT SPECT	61 patients with isolated *atypical* tremor (unilateral postural, rest o mixed tremor)	Rest tremor: abnormal DaT in 76%; of these, 75% progressed to PD after 2 years Postural tremor: abnormal DaT in 50%; of these, 50% progressed to PD after 2 years Mixed tremor: abnormal DaT in 50%; of these, 60% progressed to PD after 2 years No patient with normal baseline DaT progressed to PD after 2 years
Novellino et al. (2009) [33]	^123^I-FP-CIT SPECT ^123^I-MIBG cardiac SPECT	10 ET 22 mixed (RT + PT) tremor + mild extrapyr features 20 PD 18 HCs	ET: normal striatal and cardiac uptake in all patients PD: reduced striatal and cardiac uptake in all patients Mixed tremor: 73% reduced striatal uptake; of these, 50% reduced cardiac MIBG uptake
De Verdal et al. (2013) [30]	^123^I-FP-CIT SPECT	33 mixed tremor (RT + PT/KT)	Striatal DaT reduced in 76% of patients. No significant differences in tremor characteristics between patients with and without nigrostriatal denervation
Coria et al. (2012) [31]	^123^I-FP-CIT SPECT	167 isolated action tremor (without RT or bradykinesia)	Asymmetric tremor in 74% of patients. Positive family history in 45% of patients Visually abnormal striatal DaT in 68% of patients; of these 91% had DaT in the range of PD. Onset of tremor > 50 years and asymmetrical distribution of tremor were predictive of nigrostriatal denervation
Waln et al. (2015) [32]	^123^I-FP-CIT SPECT	22 ET “pure” 9 ET-P (ET + 1 PD cardinal sign) 8 ET + PD 13 HCs	ET “pure” and ET-P: normal DaT uptake by visual analysis. ET + PD visually abnormal Semiquantitative analysis: ET “pure” and ET-P had normal mean putamen and caudate SBR; non-significant reduction in caudate of ET compared to controls. ET-PD had reduced caudate and putamen SBR

Abbreviations:* ET*, essential tremor; *HCs*, healthy controls; *HY*, Hoehn and Yahr scale; *KT*, kinetic tremor; *PET*, positron emission tomography: *PD*, Parkinson’s disease; *PT*, postural tremor; *RT*, rest tremor; *SPECT*, single-photon emission computed tomography.

Radioligands: ^123^I-N-(3-iodopropen-2-yl)-2b-carbomethoxy-3b-(4-chlorophenyl) tropane; ^123^I-FP-CIT SPECT: ^123^I-N-3-fluoropropyl-2β-carboxymethoxy-3β-(4-iodophenyl) nortropane. ^123^I-MIBG: ^123^I-meta-iodobenzylguanidine.

A few studies have provided investigations about the state of other neurotransmitter systems in ET, such as the GABAergic and the serotonergic systems. Reduced GABAergic function in the tremor network is thought to play an important role in ET and is likely the basis of its improvement with GABAergic medication. Pathological studies have shown cerebellar degenerative changes, including a decrease in the number of GABAergic Purkinje cells [34]. ^11^C-flumazenil, a GABA_A_ receptor ligand, PET was used in one study to assess the state of the GABAergic system in 8 ET patients and 11 controls. Increased tracer binding, corresponding to an increased GABA_A_ receptor availability, was found in the ventrolateral thalamus in the ventral intermediate (Vim) nucleus, dentate nucleus and premotor cortex, sites of the tremor network. The increased GABA_A_ receptor availability is hypothesized to be a receptor upregulation process caused by a deficit in GABAergic transmission that could be due to either GABA deficiency or to the presence of abnormal and dysfunctional GABA_A_ receptor subtypes [35]. Another ^11^C-flumazenil PET study in 10 ET patients found a positive correlation between cerebellar tracer binding and tremor severity scores [36]. GABA content in the cerebellum and dentate nuclei has also been investigated with MRI spectroscopy. No significant differences were identified between 45 ET patients and 35 controls, although a significant asymmetry in GABA content was found among ET patients only [37]. Overall, only a few molecular neuroimaging studies have investigated the “GABAergic hypothesis” in essential tremor; thus, further characterization may help clarify related pathophysiological aspects.

Finally, only two small imaging studies have investigated the state of the raphe serotonergic nuclei through SERT tracers, and they did not highlight changes compared to controls or correlation to tremor scores [36, 38].

## Molecular Neuroimaging Correlates of PD Tremor

PD is characterized by rest, postural and kinetic tremors; a subtype of postural tremor, re-emergent tremor, may also be present [1, 39].

Rest tremor is the most common type of tremor in PD and thought to be generated by abnormal activity in the basal ganglia that induces tremulous activity in a cerebello-thalamo-cortical tremor network [40]. More recent evidence suggests that re-emergent tremor, i.e. a postural tremor that occurs when holding a posture for a sufficient period of time, shares the same mechanism with rest tremor [41, 42]. Molecular neuroimaging has investigated the influence of perfusion, metabolic and neurotransmitter dysfunctions, mainly dopamine, serotonin and noradrenaline, on rest tremor.

### Cerebral Blood Flow and Metabolism in PD Tremor

Perfusion abnormalities studies were among the first to investigate PD tremor. These small studies in patients with deep brain stimulation (DBS) of the ventral intermediate nucleus (Vim) of the thalamus found cerebellar blood flow increases during tremulous activity that was reversible upon successful DBS activation and subsequent tremor arrest [43–45]. Interestingly, this finding resembles cerebellar blood flow abnormalities observed in essential tremor.

The study of cerebral metabolism through FDG-PET has revealed interesting findings in PD tremulous phenotypes. PD shows a classically altered metabolic signature, named the PD-related pattern (PDRP), characterized by increased pallidothalamic and ponto-cerebellar metabolism and relative reductions in premotor and posterior parietal (associative) cortical regions [46–48]. Notably, patients with a tremulous phenotype also show a different metabolic signature named PD-related tremor pattern (PDTP) [49]. One study in nine tremor-dominant PD patients treated with Vim-DBS showed that the tremor-related pattern was characterized by metabolic increases in the cerebellum, the primary motor cortex, and to a lesser extent in the striatum [50]. This pattern was present before surgery and its expression was significantly reduced by Vim stimulation. Furthermore, the hemispheric intensity of pattern expression at baseline was associated with contralateral accelerometric measurements of tremor amplitude, and in an independent sample PDTP expression correlated with UPDRS tremor scores. This observation suggests that tremor itself, and not just the tremulous phenotype, is related to the expression of the tremor-related network. A subsequent multitracer study with FDG and ^18^FDOPA PET showed that higher dopaminergic terminal availability in the striatum of tremor-dominant patients was also associated with a higher glucose metabolic rate compared to akinetic-rigid patients [51]. Overall, metabolic imaging in PD has provided evidence that specific alterations may be identified between different phenotypes, that a metabolic signature, i.e. the PD tremor-related pattern, is typical of tremulous PD, and that the expression of this signature is also associated with tremor severity. A summary of the studies investigating metabolic and other non-dopaminergic correlates of parkinsonian tremor is shown in Table 3.

**Table 3:** Summary of selected studies investigating non-dopaminergic correlates of PD tremor

Author, year	Technique	Cohort	Findings
PET-FDG metabolic investigations			
Antonini et al. (1998) [49]	^18^FDG PET	8 PD with and 8 without tremor in off-state 10 HCs	PD with tremor had increased metabolic rate in a network comprising the thalamus, pons, and premotor cortical regions, a network that was called the PD related tremor pattern (PDTP) While all patients with PD usually express metabolic alterations in a metabolic network called PDRP, tremulous phenotype also require the expression of the PDTP
Lozza et al. (2002) [89]	^18^FDG PET	17 non-demented PD in the ON state	Bradykinesia scores positively correlated with putaminal and pallidal metabolic rates. Tremor scores negatively correlated with putamen and cerebellar vermis metabolic rate
Mure et al. (2011) [50]	^18^FDG PET	9 tremor dominant PD patients scanned at baseline and during Vim-DBS	Baseline metabolic pattern revealed a PD tremor-related network composed of cerebellum/dentate nuclei, primary motor cortex and striatum. Vim stimulation resulted in consistent reductions in this pattern expression. Without stimulation, pattern expression values correlated with accelerometric measurements of tremor amplitude
Eggers et al. (2014) [51]	^18^F-DOPA ^18^FDG PET	64 PD patients (32 TD)	Higher putamen and caudate metabolism in tremulous PD vs akinetic-rigid PD. Striatal ^18^F-DOPA and FDG activity were closely correlated
Serotonergic pathways investigations			
Doder et al. (2003) [69]	^11^C-WAY 100,635 PET (5HT_1A_ receptor ligand)	23 PD 8 HCs	UDPRS total and rest tremor scores, but not bradykinesia and rigidity, were inversely correlated with tracer binding in midbrain raphe
Caretti et al. (2008) [90]	^123^I-beta-CIT (SERT ligand)	32 PD (23 scanned also after 17 months) 13 HCs	PD with tremor have significantly reduced uptake in thalamus, representing mostly SERT availability, compared to PD without tremor; no differences at follow up (indicating greater declines in PD without tremor)
Loane et al. (2013) [72]	^11^C-DASB PET (SERT ligand)	12 TD-PD 12 AR-PD 12 HCs	^11^C-DASB uptake in putamen, caudate and raphe nuclei was negatively correlated with severity of action tremor, but not with rest tremor in TD-PD. TD-PD also showed SERT reductions in caudate and putamen compared to AR-PD
Qamhawi et al. (2015) [70]	^123^I-FP-CIT SPECT (SERT ligand)	345 de novo PD 145 HCs	^123^I-FP-CIT raphe uptake, representative of SERT availability, was inversely associated with rest tremor severity in the whole cohort and in a subgroup of tremulous patients
Pasquini et al. (2018) [71•]	^123^I-FP-CIT SPECT (SERT ligand)	378 de novo PD 145 HCs	In TD patients rest tremor severity was inversely associated with raphe SERT at baseline and after two years. More severe raphe dysfunction coupled with less severe putaminal dopaminergic denervation was associated with higher rest tremor scores and reduced rest tremor response to dopaminergic therapy
Noradrenergic pathways investigations			
Isaias et al. (2011) [74]	^123^I-FP-CIT SPECT	82 PD	^123^I-FP-CIT binding in pons, representing locus coeruleus noradrenergic transporter, was significantly higher in PD patients than controls. No differences between patients with and without tremor
Nahimi et al. (2018) [75]	^11^C-MeNER PET (NET ligand)	15 PD 10 HCs	Tracer uptake significantly reduced in red nucleus and thalamus of PD patients. Tremor occurrence was associated with higher thalamic tracer binding in PD. Tremor severity not associated with tracer binding in any region
Kinnerup et al. (2021) [76•]	^11^C-MeNER PET (NET ligand)	65 PD (28 tremulous) 28 HCs	Tracer uptake was significantly higher in LC and thalamus of tremulous patients. In tremulous patients LC tracer uptake was similar to controls, while in the raphe was significantly reduced. In non-tremulous patients, tracer uptake was significantly reduced in all regions compared to controls Rest tremor severity was associated with higher NET availability in median raphe, thalamus and red nuclei

Abbreviations:* AR*, akinetic-rigid; *HCs*, healthy controls; *NET*, noradrenaline transporter; *PD*, Parkinson’s disease; *PET*, positron emission tomography; *SERT*, serotonin transporter; *SPECT*, single-photon emission tomography; *UPDRS*, Unified Parkinson’s Disease Rating Scale; *TD*, tremor dominant

Radioligands: ^18^FDG: ^18^F-fluorodeoxyglucose; ^18^F-DOPA: ^18^F-fluoro-L-dopa; ^123^I-beta-CIT SPECT: ^123^I-2β-carbomethoxy-3β-(4-iodophenyl)tropane; ^123^I-FP-CIT SPECT: ^123^I-N-3-fluoropropyl-2β-carboxymethoxy-3β-(4-iodophenyl) nortropane; ^11^C-WAY 100,635: ^11^C-(N-[2-]4-(2-methoxyphenyl)-1-piperazinyl]ethyl]-N-(2- pyridinyl)cyclohexanecarboxamide); ^11^C-DASB: ^11^C-3-amino-4-(2-dimethylaminomethyl-phenylsulfanyl)benzonitrile; ^11^C-MeNER: ^11^C-methylreboxetine

### Receptor Imaging in PD Tremor

Neurotransmitter dysfunction has been extensively investigated in PD tremor especially through dopaminergic imaging. Nigral degeneration and associated striatal dopaminergic loss are the hallmark of PD and thought to be necessary for the generation of PD tremor. However, as highlighted in the previous paragraph, phenotypic expression of rest tremor may be sometimes unrelated to nigrostriatal degeneration, as it is in some cases of essential tremor with rest tremor. A further complication in establishing the pathophysiology of PD tremor is the fact that its clinical severity seems unrelated to the degree of nigrostriatal denervation, as opposed to bradykinesia and rigidity [52–61]. In one study, accelerometer-based measurement of tremor severity failed to find an association with contralateral striatal DaT [62]. However, the index of asymmetry of tremor power and striatal DaT were associated, indicating a relationship between higher tremor severity and nigrostriatal denervation in the more affected hemisphere. A recent study has employed surface EMG and tri-axial accelerometers in order to establish whether a more precise quantification of tremor may reflect dopaminergic denervation [63]. A series of recordings in various settings (e.g. at rest, posturing, index-to-nose movements, with and without mental or motor distraction) was carried out and, quite strikingly, tremor power recorded by different sensors in many resting conditions were correlated with contralateral striatal DaT availability. It must be mentioned that total UDPRS III tremor scores also correlated with contralateral striatal uptake, a finding that is in contrast with many previous studies. Therefore, we cannot exclude that this finding is specifically related to the cohort studied and needs replication in different and larger cohorts. Nonetheless, a more precise evaluation of tremor, such as that carried out by Fois and colleagues, may reveal previously undetected findings. Although in previous studies severity of nigrostriatal denervation and tremor were not associated, differences in tremor-dominant (TD) and akintic-rigid (AR) patients could be identified. Indeed, it is a common finding that patients with a tremulous phenotype have a significantly more preserved striatal DaT availability [64, 65]. It has also been shown that a different pattern of nigrostriatal denervation may be visually spotted in the two phenotypes: in one study Eggers and colleagues showed that scans of TD patients are more frequently associated with an “eagle wing” shape of striatal DaT availability [66]. This pattern resembles that of pathological descriptions of tremulous patients with a higher degree of degeneration in the dopaminergic retrorubral area (A8) that projects to the lateral putamen[67]. Pallidal dopaminergic denervation has also been hypothesized to be involved in tremor generation. In a seminal paper on tremor pathophysiology, Helmich and colleagues used a multimodal approach to simultaneously study basal ganglia dopaminergic denervation, the cerebello-thalamo-cortical tremor network and quantitative tremor assessment [68]. In tremulous patients, pallidal DaT was lower in the most affected hemisphere compared to non-tremulous patients, and rest tremor severity correlated with pallidal, but not striatal, DaT. With fMRI, they also showed that while the basal ganglia were activated transiently at the onset of tremor episodes, activity in the cerebello-thalamo-cortical circuit covaried with tremor amplitude. Therefore, it was hypothesized that dopaminergic dysfunction in the basal ganglia is capable of driving the cerebello-thalamo-cortical circuit into tremor activity, in a “switch and dimmer” fashion.

The lack of correlation between nigrostriatal denervation and rest tremor severity, coupled with mixed responses to dopaminergic medication, has sparked research into non-dopaminergic determinants of PD tremor, especially serotonin and noradrenaline. A first PET report on the serotonergic influence on tremor showed that a serotonin receptor (5HT_1A_) ligand uptake in the midbrain raphe correlated inversely with total and rest tremor scores, but not with bradykinesia and rigidity [69]. It was hypothesized that degeneration of the serotonergic raphe nuclei projections could modulate the expression of tremor in PD. In two subsequent studies on the large cohort of the de novo PD patients from the Parkinson’s Progressive Markers Initiative (PPMI), median raphe serotonin transporter (SERT) availability, assessed with ^123^I-FP-CIT SPECT, showed inverse correlations with tremor severity in the entire cohort of PD patients and in the tremulous subgroup [70, 71•]. Furthermore, it was also shown that more severe raphe dysfunction, coupled with less severe dopaminergic denervation (i.e. a low raphe/putamen ratio), was associated with higher tremor scores and worse responses to dopaminergic medication [71•]. Therefore, while dopaminergic denervation appears necessary for rest tremor generation, the level of serotonergic dysfunction could modulate the intensity of tremor and its response to dopaminergic medication. It has also been suggested that the serotonergic system may modulate action tremor in PD. In a PET study involving 12 tremulous and 12 non-tremulous PD patients, it was shown that SERT availability in the putamen, caudate and raphe nuclei was inversely associated with the severity of action tremor, but not of rest tremor. This discrepancy on rest tremor compared to other studies may be due to the smaller number of patients included in the study that may have been underpowered to detect such association[72].

Noradrenergic dysfunction has been shown to influence tremor in PD. Central noradrenergic projections originate from the pontine locus coeruleus, known to be activated under stressful conditions. These are known in turn to exacerbate tremor [73]. Molecular neuroimaging of the noradrenergic system is a recent clinical research advancement and only a few studies have been carried out. One study investigated locus coeruleus noradrenergic transporter (NET) availability through ^123^I-FP-CIT SPECT but failed to show a relationship between locus coeruleus imaging abnormalities and tremor [74]. More recent PET investigations using ^11^C-methylreboxetine (11C-MeNER), a selective NET ligand, have highlighted different aspects of noradrenergic dysfunction in PD. It was shown that tremulous patients had significantly higher tracer uptake in the locus coeruleus and thalamus compared to non-tremulous patients. Tremulous patients had a locus coeruleus uptake similar to controls whereas the raphe was the only site with a significant reduction. Furthermore, rest tremor scores were positively associated with median raphe, thalamus and red nucleus tracer uptake [75, 76•]. Like neuropathological studies, these reports indicate that tremulous patients have a lower pathologic burden in the locus coeruleus compared to akinetic-rigid patients. It is interesting to note that the only binding site with reduced NET availability, and thus noradrenergic terminals, in tremulous patient was the median raphe, and that NET availability in the median raphe was associated with rest tremor severity. It is tempting to think that an interaction between the noradrenergic and the serotonergic ascending systems, especially in the tremor dominant form of PD, could modulate the expression of tremor. Multimodal and multitracer neuroimaging studies may help further clarify this issue.

Overall, molecular neuroimaging studies investigating PD tremor have helped clarify many pathophysiological aspects. One caveat in interpreting some of these studies is that tremulous phenotypes, rather than tremor itself, were investigated. Therefore, while many studies did establish associations with tremor severity, thus likely indicating a direct influence on tremor itself, others described associations with a specific phenotype. The latter must be interpreted with caution in terms of tremor pathophysiology, since abnormalities may be due to the phenotype itself, which may in turn be due a specific neuropathological pattern, rather than being directly associated with tremor.

## Other Types of Tremor

### Dystonic Tremor

Tremor may be a clinical feature associated with dystonia, and dystonia often presents with tremor. Furthermore, isolated tremor syndromes such as isolated head tremor may be forms of dystonia without clear abnormal posturing [77–79]. A recent study assessed striatal DaT availability in 14 patients with isolated head tremor and 14 controls [80•]. While all patients were found to have abnormal somatosensory temporal discrimination threshold (STDT), a feature usually associated with dystonia, normal dopaminergic transmission was documented. No correlation between nigrostriatal function and STDT was found, suggesting that neurophysiological measures associated with dystonia are not influenced by striatal DaT availability in this tremor syndrome. Conversely, the presence of striatal D2/D3 receptor abnormalities has been hypothesized in dystonia but it still subject to ongoing debate [81, 82].

### Holmes Tremor

Holmes tremor is a syndrome of low frequency (< 5 Hz) rest, postural and kinetic tremor that involves proximal and distal muscles [1]. It is generally thought as a network disorder involving lesions in the cerebello-thalamo-cortical or dentate-rubro-olivary pathways, and in the dopaminergic striatal system [83]. A recent study on the human brain connectome identified lesions causing Holmes tremor in a network that includes the red nucleus, internal globus pallidus, thalamus (ventralis oris posterior and pulvinar), cerebellum (vermis, lateral cerebellar cortex and flocculonodular) and the pontomedullary junction [84]. Another study revealed tremor amplitude-related activity in the sensorimotor cortex and cerebellar vermis in a patient with Holmes tremor caused by a microbleed near the right red nucleus that caused ipsilateral nigrostriatal dopaminergic denervation [85]. Nigrostriatal dopaminergic denervation is a variable finding in Holmes tremor and does not always correspond to a good levodopa responsiveness [83, 85–87]. Therefore, it has been suggested that lesions causing Holmes tremor do not localize to any single region, but instead localize to a functionally connected network [84]. The regional heterogeneity of the lesions causing Holmes tremor partially explains why some patients do not show nigrostriatal denervation or levodopa responsiveness. However, it is currently unknown how dopaminergic dysfunction influences the pathophysiology of Holmes tremor and the predictive factors of levodopa responsiveness. Multimodal neuroimaging studies including nigrostriatal dopaminergic imaging could help clarify this issue and select patients for the appropriate treatment, either pharmacological or surgical.

## Conclusions

Tremor is a common hyperkinetic movement disorder with a complex pathophysiology. The most frequent tremor causes are essential tremor and Parkinson’s disease tremor. A shared pathophysiological substrate of tremor likely lies in a cerebello-thalamo-cortical network capable of generating tremor oscillations and ultimately the involuntary movements. Neurotransmitter systems outside this network, such as dopamine, noradrenaline and serotonin, are able to influence the clinical manifestation of tremor. Molecular neuroimaging has provided great insights into the clinical correlates of tremor in ET and PD. In ET, cerebral blood flow and metabolic abnormalities have been characterized and they have demonstrated potential for translation into useful therapeutic biomarkers [16•, 17•]. GABA_A_ receptor abnormalities have also been found and shown to be associated with tremor severity [35, 36]. Conversely, studies investigating the state of the noradrenergic system are lacking. The recent availability of new noradrenaline transporter tracers that have already been used in PD may help clarify the influence of the noradrenergic system in ET. The dopaminergic system is not involved in classical ET, but may be degenerated in some isolated tremor syndromes with atypical features, and its investigation may thus be useful in establishing a differential diagnosis with PD. It is thought that ET patients may have an increased risk of PD, but no predictive biomarkers exist in the absence of initial nigrostriatal denervation.

Rest tremor is one of the hallmarks of PD and it is thought to arise from basal ganglia dopaminergic dysfunction and its influence on the cerebello-thalamo-cortical network, a dysfunctional system that is thought to work in a “switch and dimmer” fashion [40]. The degeneration of the serotonergic and noradrenergic systems are also known to influence the manifestation of tremor in PD [68–70, 71•, 87]. Interestingly, tremulous PD also shows a typical metabolic signature, called the PD related tremor pattern, with increased glucose metabolic rates in cerebellum/dentate nucleus and primary motor cortex, and, to a lesser extent, the striatum [50].

Over the past few years, the pathophysiology of tremor has become increasingly clearer. However, both in ET and PD available medications may not be efficacious in all patients.

Therefore, there is a great need to transform an increasing wealth of knowledge about the mechanisms of tremor into new treatments.
